# A Novel Inflammation-Related Gene Signature for Overall Survival Prediction and Comprehensive Analysis in Pediatric Patients with Wilms Tumor

**DOI:** 10.1155/2022/2651105

**Published:** 2022-05-07

**Authors:** Jiahao Zhang, Yongchang Lai, Langjing Zhu, Zechao Lu, Chuxian Hu, Haobin Zhou, Zeguang Lu, Zhicheng Tang, Zhaohui He, Fucai Tang

**Affiliations:** ^1^The Sixth Affiliated Hospital of Guangzhou Medical University, Qingyuan, Guangdong 511500, China; ^2^Department of Urology, The Eighth Affiliated Hospital, Sun Yat-sen University, Shenzhen, Guangdong 518033, China; ^3^Department of Nephrology, The Eighth Affiliated Hospital, Sun Yat-sen University, Shenzhen, Guangdong 518033, China; ^4^The First Clinical College of Guangzhou Medical University, Guangzhou, Guangdong 511436, China; ^5^The Second Clinical College of Guangzhou Medical University, Guangzhou, Guangdong 511436, China; ^6^The Third Clinical College of Guangzhou Medical University, Guangzhou, Guangdong 511436, China

## Abstract

Wilms tumor (WT) is a common pediatric renal cancer, with a poor prognosis and high-risk recurrence in some patients. The inflammatory microenvironment is gradually gaining attention in WT. In this study, novel inflammation-related signatures and prognostic model were explored and integrated using bioinformatics analysis. The mRNA profile of pediatric patients with WT and inflammation-related genes (IRGs) were acquired from Therapeutically Available Research to Generate Effective Treatments (TARGET) and Gene Set Enrichment Analysis (GSEA) databases, respectively. Then, a novel prognostic model founded on 7-IRGs signature (BICC1, CSPP1, KRT8, MYCN, NELFA, NXN, and RNF113A) was established by the least absolute shrinkage and selection operator (LASSO) and multivariate Cox regression to stratify pediatric patients with WT into high- and low-risk groups successfully. And a stable performance of the prognostic risk model was verified in predicting overall survival (OS) by receiver-operating characteristic (ROC) curves, Kaplan-Meier (KM) curves, and independent prognostic analysis (*p* < 0.05). In addition, a novel nomogram integrating risk scores with good robustness was developed and validated by *C*-index, ROC, and calibration plots. The potential function and pathway were explored via Gene Ontology (GO), Kyoto Encyclopedia of Genes and Genomes (KEGG), and GSEA, with mainly inflammation and immune-related biological processes. The higher-risk scores, the lower immune infiltration, as shown in the single-sample GSEA (ssGSEA) and tumor microenvironment (TME) analysis. The drug sensitivity analysis showed that regulating 7-IRGs signature has a significant correlation with the chemotherapy drugs of WT patients. In summary, this study defined a prognostic risk model and nomogram based on 7-IRGs signature, which may provide novel insights into clinical prognosis and inflammatory study in WT patients. Besides, enhancing immune infiltration based on inflammatory response and regulating 7-IRGs signature are beneficial to ameliorating the efficacy in WT patients.

## 1. Introduction

Wilms tumor (WT), known as nephroblastoma, accounts for 5% of all pediatric patients with the tumor and 75% of pediatric patients with WT between 1- and 5-year-old [1]. The 5-year relative survival for WT patients under 14-year was 93.2%, but patients with diffuse anaplastic Wilms tumor (DAWT) had a poor 4-year relapse-free survival most up to 40% only [2, 3]. Currently, many factors affect the risk assessment of WT patients, such as tumor stage and histology, molecular markers (LOH of 16q and1p), and clinical characteristics [4]. Risk assessment in WT patients facilitates the treatment of long-term toxicity and minimizes the risk of recurrence, as well as determining the need for postoperative adjuvant therapy, of which molecular markers are regarded as the factor to determine the final risk assessment [1]. In recent years, although 32 biomarker markers were identified in WT patients [5], it is necessary to further enrich the potential biological prognostic signature based on the biological metabolism process and excavate more effective prognostic scoring models.

The metabolic process of inflammatory response is considered one of the rings in the tumor microenvironment (TME) [6]. There are a lot of related factors and pathways to the inflammation response in TME, such as tumor macrophages (TAMs), dendritic cells, lymphocytes, proinflammatory cytokines, nuclear factor-*κ*B (NF-*κ*B), and c-Jun N-terminal kinase (JNK), which impacts the progression of the tumor [7]. And inflammation plays a dual role in promoting tumor initiation and inhibiting tumor development [8]. Some studies pointed out explicitly that acute inflammation can resist tumor development by the enhancement of antitumor immunity, while chronic inflammation promotes tumorigenesis by providing an ideal growth microenvironment [6, 9]. And TAMs were the main type of infiltrating cells in the inflammatory microenvironment of WT patients, and more inflammation-related proteins and cytokines were identified gradually by exact experiments, such as COX-2, VEGF, Trx1, and HIF-1 [10–12]. And COX-2 inhibitors to decrease tumor cell growth become a probability in WT [13]. The inflammatory response is considered to be closely related to anticancer therapy in these factors of the TME [14]. Currently, the treatment of WT patients is principally neoadjuvant chemotherapy, surgical resection, and postoperative chemotherapy [15]. A previous study indicated that macrophage migration inhibitory factor (MIF) and CXCL7 as tumor proinflammatory factors were identified by proteomics to correlate with clinical stage and development in WT patients [11]. And the inflammatory pseudocapsule was considered one of relapse-associated factors in WT [16]. In summary, these pieces of evidence support the existence and influence of the inflammatory microenvironment in WT. Recently, the inflammatory response is interesting biological processing to probe diversified prognostic models and potential value in cancer treatment and has been applied in more and more cancers, such as bladder cancer, hepatocellular carcinoma, and pancreatic ductal adenocarcinoma [17–19]. However, there are not many studies available for the observation in prognosis and the target treatment-based inflammatory response of WT patients. It is necessary to develop a novel prognostic model based on inflammation-related mRNA to explore more potential and effective targets from the collection of these genes about inflammation-related proteins and cytokines using bioinformatics analysis in pediatric patients with WT.

In this study, a novel prognostic risk model of seven inflammation-related signatures was identified to stratify pediatric patients with WT into high- and low-risk groups based on risk scores using bioinformatics analysis. Combined with clinical characteristics, this study constructed a collective nomogram model integrating risk scores. In addition, through exploring immune infiltration and TME scores, the differences of immune status between the two risk groups were analyzed further. Drug sensitivity analysis showed the connection of signatures and drug activity to reveal the potential treatment strategy in pediatric patients with WT. In summary, the risk model is an independent prognostic factor, and the inflammation-related prognostic signature can become a potential treatment direction for pediatric patients with WT.

## 2. Materials and Methods

### 2.1. Data Acquisition

The mRNA sequencing profile and corresponding clinical information of WT were downloaded from Therapeutically Applicable Research to Generate Effective Treatments (TARGET, https://ocg.cancer.gov/programs/target/data-matrix) on August 1, 2021, a database that is aimed at improving pediatric cancer treatments. These basic clinical characters involving gender, age, tumor stage, endpoint event, histologic, overall survival time, and status must be included. Averaging the repeated samples and excluding samples without overall survival, 125 tumor samples and 6 control samples based on adjacent normal tissue were used in our study. The data set of RPKM (reads per kilobase of transcript per million reads mapped) was converted to TPM (transcripts per million) by the following formula: TPM = (10^6^∗RPKM)/sum(RPKM) [20]. Four gene sets about the inflammation-related response, M38152, M5932, M17322, and M39641, were selected from the molecular signature database of Gene Set Enrichment Analysis (GSEA, http://www.gsea-msigdb.org/gsea/msigdb/index.jsp) on August 2021. The additional notes about inflammation-related genes were shown in Table S1. Ultimately, a total of 1137 inflammation-related genes (IRGs) were applied in our study through data deduplication and intersection with the mRNA profile of WT patients.

### 2.2. Differential Expression of Inflammation-Related Prognostic Genes

Firstly, the R package “limma” was utilized to identify the differentially expressed inflammation-related genes (DE-IRGs) with the certification requirement of |log_2_ [Fold Change (FC)]| >1 and false discovery rate (FDR) < 0.05 between 125 tumor samples and 6 control samples. Secondly, overall survival (OS) and survival status were associated with the IRGs expression among 125 WT samples using univariate Cox regression analysis to identify OS-related genes by the R package “survival” with *p* < 0.05. These inflammation-related prognostic genes (IRPGs) were intersected between DE-IRGs and OS-related genes through the “Venn” R package. When the volcano and heatmap were plotted, the distribution of DE-IRGs and IRPGs was shown, respectively. The hazard ratio (HR) with 95% confidence interval (CI) of IRPGs was calculated by the R package “survival,” using forest plots to show. The correlation of IRPGs was plotted by the “corplot” package based on the coefficients of each both IRPGs.

### 2.3. Develop an Inflammation-Related Prognostic Gene Model

To identify risk IRPGs through reducing the multicollinearity effect between IRPG expression and OS among pediatric patients with WT, the Least Absolute Shrinkage and Selection Operator (LASSO) regression was applied by the R package “glmnet.” Tenfold crossvalidation processing to select the optimal lambda value was carried out with the minimum partial likelihood deviance. Narrowing the range of risk signatures to develop a credible IRPG risk model, a multivariate Cox regression was used to seek optimal risk signatures by the lowest value of Akaike information criterion (AIC) and calculate their coefficients and risk scores of pediatric patients with WT. The formula of risk scores used for the model was as follows:

(1) $$\begin{array}{c} \text{RISKSCORE}=\overset{n}{\underset{i=1}{\sum }}\left(\text{Coef}\times \text{Exp}\right), \end{array}$$

where *n*, Coef, and Exp represented the number of genes, regression coefficients, and expression of the related genes obtained from multivariate Cox regression, respectively. And pediatric patients with WT were segregated into high- and low-risk groups according to the median value of risk scores in all WT samples. Afterward, the “survival” package was utilized to analyze the OS of two risk groups by plotting the Kaplan-Meier (KM) curves with a log-rank test. The time-dependent receiver-operating characteristic curves (ROC) were utilized to validate the predictive power of the prognostic model by the “timeROC” package. In addition, *t*-distributed stochastic neighbor embedding (t-SNE) and principal component analysis (PCA) were performed using the R package “Rstne” to contour the expression pattern of WT samples and visualize whether the high- and low-risk samples could be distinguished via dimensionality reduction.

### 2.4. Clinical Characteristics Relevance Analysis

With clinical features including age, endpoint event, stage, gender, and histologic, chi-square analysis or fisher's exact probability test was used between two risk groups. Furthermore, the relevance between clinical characteristics and risk scores was analyzed in all pediatric patients with WT using the wilcox test of R package “limma.” To evaluate the independent survival predictive performances of clinical characteristics and risk scores, univariate and multivariate Cox regression analyses were carried out using the R package “survival.”

### 2.5. Construction of a Nomogram Integrating Clinical Characteristics

Based on the significant factors from univariate Cox regression analysis (*p* < 0.05) in the independent prognosis analysis, a series of clinical information was determined to generate a novel nomogram integrating risk score for predicting OS by multivariate Cox regression. To assess the prediction ability of the nomogram, the bootstrap method was performed to calculate the concordance index (*C*-index) corrected by 1,000 resamples. The ROC curves were plotted for the purpose as same as the *C*-index. In addition, calibration curves were used to describe the consistency between the nomogram-predicted risks and the actual risks at the 1-, 3-, and 5-year survival rates.

### 2.6. Functional Enrichment Analysis and Tumor Microenvironment Analysis

The Gene Ontology (GO) and the Kyoto Encyclopedia of Genes and Genomes (KEGG) analysis were applied to DE-IRGs between control and tumor samples using the R package “clusterprofiler.” To show further enrichment of biological functions and pathways between high- and low-risk groups, the GO and KEGG gene sets were downloaded from gene set enrichment analysis (GSEA) (http://www.gsea-msigdb.org/gsea/downloads.jsp) and performed using the R package “clusterprofiler” and *p* < 0.05 to screen significant functions and pathways [21]. In addition, the single-sample gene set enrichment analysis (ssGSEA) was used to assess immune-related scores and probe differences of immune-related cells and pathways between high- and low-risk groups by the R package “GSVA” and “GSEABase.” To evaluate the relationship between cell content and risk score of the TME in WT patients, the ESTIMATE algorithm was used to score the content of stromal cells and immune cells in WT patients using the R package “estimate” [22]. Through the Spearman method, the visualization of the correlation between TME scores and risk scores was plotted.

### 2.7. Drug Sensitivity Analysis

The CellMiner database (https://discover.nci.nih.gov/cellminer/home.do) was designed for the study to the relationship of efficacy among NCI-60 cancerous cell lines, chemical compounds, and natural products [23]. The CellMiner database was used to excavate the potential correlation between inflammation-related signatures and antitumor drugs approved by American Food and Drug Administration (FDA) in pediatric patients with WT. The *Z* scores associated with drug activity expressed as 50% growth inhibitory levels (GI50s) and RNA-seq expression of NCI-60 were downloaded from the CellMiner database. Then, the “limma” package was used to explore the potential correlation using Pearson's correlation analysis with *p* < 0.05. A total of R packages was operated in R software (version 4.1.0).

## 3. Results

### 3.1. Identification of Prognostic DE-IRGs

**Figure 1:**
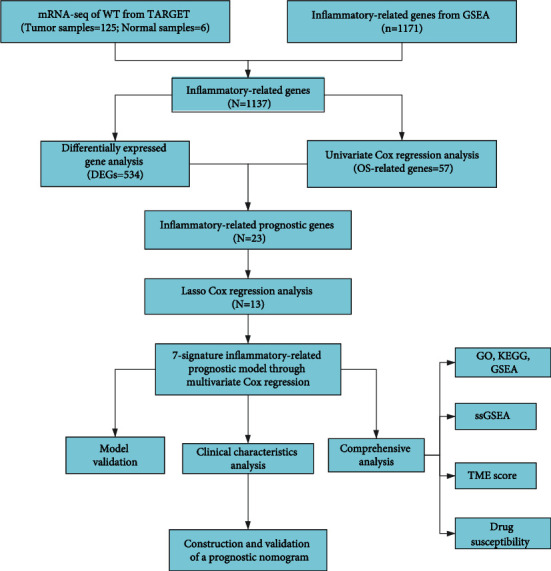
The workflow chart of this study processes.

**Figure 2:**
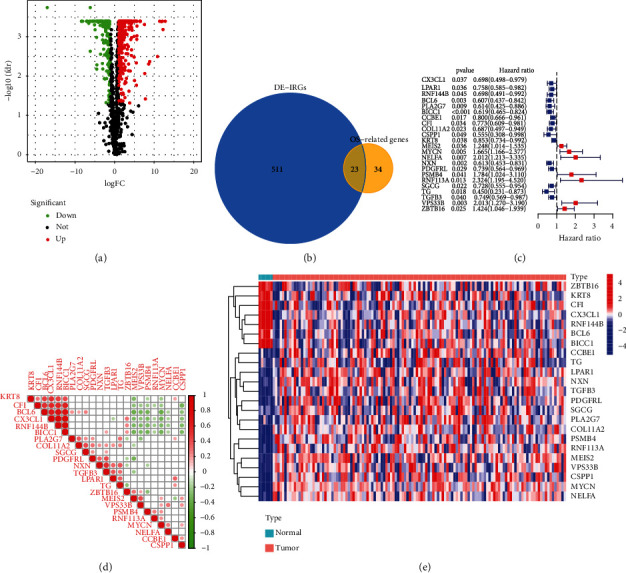
Identification of DE-IRGs and IRPGs. (a) Volcanic map for displaying the up- and downregulation of DE-IRGs. (b) Venn plotting IRPGs overlapped between DE-IRGs and OS. (c) Forest plots to show the results of the univariate Cox regression analysis between IRPGs expression and OS. (d) The relevance heatmap revealed the correlation among IRPGs. (e) The heatmap showed the differences of IRPGs between tumor and normal tissues.

The flowchart of the study process was presented in Figure 1. The 534 DE-IRGs were identified from 1137 IRGs between 125 WT samples and 6 control samples, including 342 upregulated genes and 192 downregulated genes (Figure 2(a)). Based on univariate Cox regression analysis between OS and the expression of IRGs, 57 OS-related IRGs were identified (*p* < 0.05). Then, 23 IRPGs were screened from the intersection between DE-IRGs and OS-related genes (Figure 2(b)). A total of 11 IRPGs were regarded as high-risk IRPGs with the HR > 1, while the remaining IRPGs were protective factors with HR < 1 (Figure 2(c)). The positive and negative correlation among 23 IRPGs was shown in Figure 2(d). A heatmap was plotted to show the distribution of 23 IRPGs between WT and control samples, which indicated that 7 IRPGs were downregulated in WT samples, and the other 16 IRPGs were downregulated in control samples (Figure 2(e)).

### 3.2. Establishment and Assessment of the 7-IRPGs Signature

**Figure 3:**
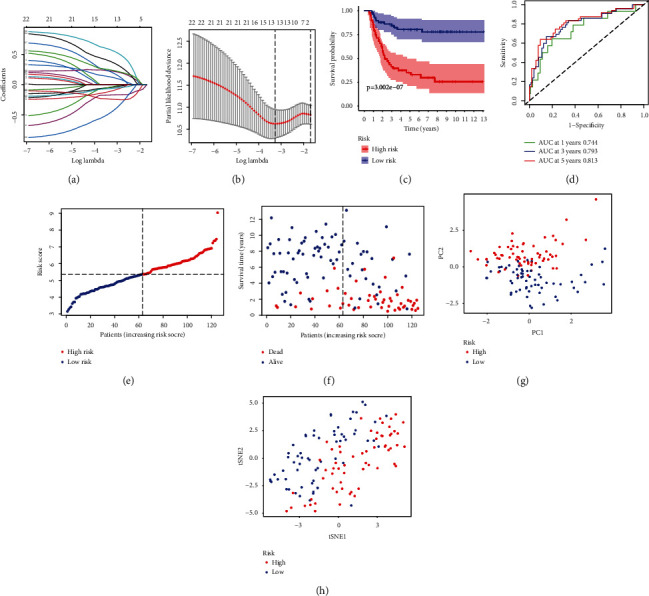
Construction of the inflammation-related prognostic signatures using LASSO regression analysis. (a, b) LASSO regression analysis among 23 IRPGs. Each curve corresponds to an IRPG in the LASSO model. Partial likelihood deviance with 10-fold crossvalidation tuning the parameter selection was used to screen the best lambda. Upper *X*-axes were the number of included IRPGs, while lower *X*-axes were log lambda (*λ*) whose greater, the greater the punishment of the linear model. (a) *Y*-axes mean coefficients in each IRPGs; (b) *Y*-axes was partial likelihood deviance values that mean the magnitude of the error included in the variable model. (c) Kaplan–Meier curves for the 7-IRGs signature relative to OS outcomes of patients in the high- and low-risk groups. (d) AUC of the ROC curves demonstrated the predictive efficiency of the 7-IRGs signature model. (e, f) The distribution of pediatric patients with WT and survival status was based on the risk scores in the low- and high-risk groups, and the dotted line was the median value of risk scores (g, h) PCA plot and t-SNE analysis with dimension reduction.

The more significant genes of 14 IRPGs were screened from 23 IRPGs using LASSO regression based on the optimal lambda value (Figures 3(a) and 3(b)). Then, 7-IRPGs signature (BICC1, CSPP1, KRT8, MYCN, NELFA, NXN, and RNF113A) was identified through multivariate Cox regression in 14 IRPGs, which would be applied for the establishment of a prognostic risk model in pediatric patients with WT using the coefficient of 7-IRPG signature. The risk score of each WT patient was counted as follows: risk score = (−0.466158 × the expression level of BICC1) + (−0.481445 × the expression level of CSPP1) + (−0.119206 × the expression level of KRT8) + (0.324931 × the expression level of MYCN) + (0.719828 × the expression level of NELFA) + (−0.315503 × the expression level of NXN) + (0.914112 × the expression level of RNF113A). Based on the median risk scores that were calculated from the coefficient of signature and expression level of each sample, 125 pediatric patients with WT were divided into a high-risk group (*n* = 62) and a low-risk group (*n* = 63) (Figures 3(e) and 3(f)). The pediatric patients with WT in the high-risk group had a lower survival rate than those in the low-risk group (Figure 3(c)). According to ROC curves, demonstrating a stable performance of the 7-IRPGs signature risk model, the areas under the ROC curves (AUCs) were 0.744, 0.793, and 0.813 for the WT patients at 1-, 3-, and 5-year OS, respectively (Figure 3(d)). The pediatric patients with the high- and low-risk groups were centered successfully in two directions using PCA and t-SNE analysis methods according to expression levels of 7-IRPGs signature (Figures 3(g) and 3(h)).

### 3.3. Clinical Characteristics and Independent Prognostic Analysis

**Figure 4:**
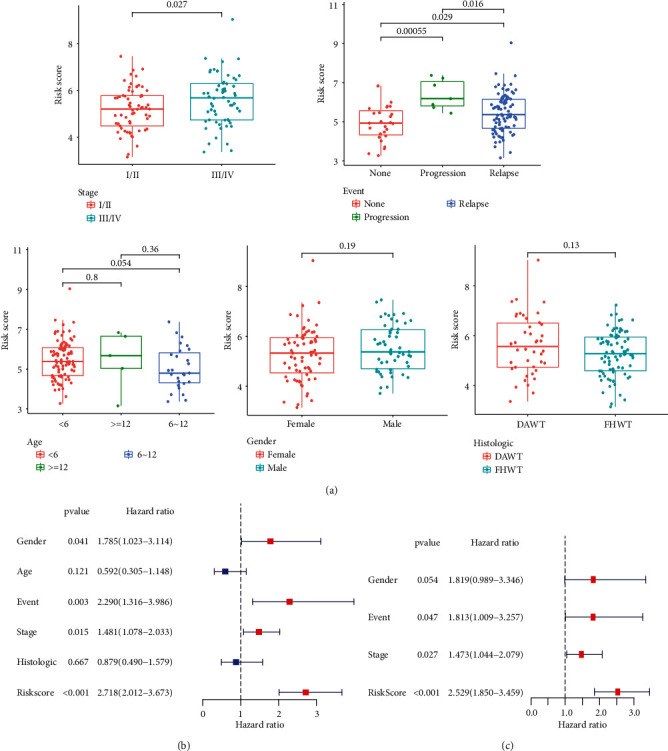
Relevance analysis of clinical characteristics. (a) The differences of clinical characteristics in pediatric patients with WT, including stage, events, age, gender, and histology. (b, c) Univariate and multivariate regression analysis outcomes of the relationships between the OS and the clinical parameters.

The clinical information of high- and low-risk groups was shown in Table 1. The differences between risk scores and clinical characteristics analyzed further in our study were shown in Figure 4(a). The stage and endpoint event of pediatric patients with WT were significantly differentiated in risk scores (Figure 4(a)). The univariate Cox regression analysis showed that gender [HR: 1.785, 95% CI (1.023-3.114), *p* = 0.041], event [HR: 2.290, 95% CI (1.316-3.986), *p* = 0.003], stage [HR: 1.481, 95% CI (1.078-2.033), *p* = 0.015], and risk scores [HR: 2.718, 95% CI (2.012-3.673), *p* < 0.001] had significant differences (Figure 4(b)). The factors significantly differentiated in the univariate Cox regression analysis were involved in the multivariate Cox regression analysis, which demonstrated that event, stage, and risk scores also had significant differences so that risk scores [HR: 2.529, 95% CI (1.850-3.459), *p* < 0.001], event [HR: 1.813, 95% CI (1.009-3.257), *p* = 0.047], and stage [HR: 1.473, 95% CI (1.044-2.079), *p* = 0.027] can be regarded as an independent prognostic factor in pediatric patients with WT (Figure 4(c)).

### 3.4. Construction and Evaluation of a Prognostic Nomogram

**Figure 5:**
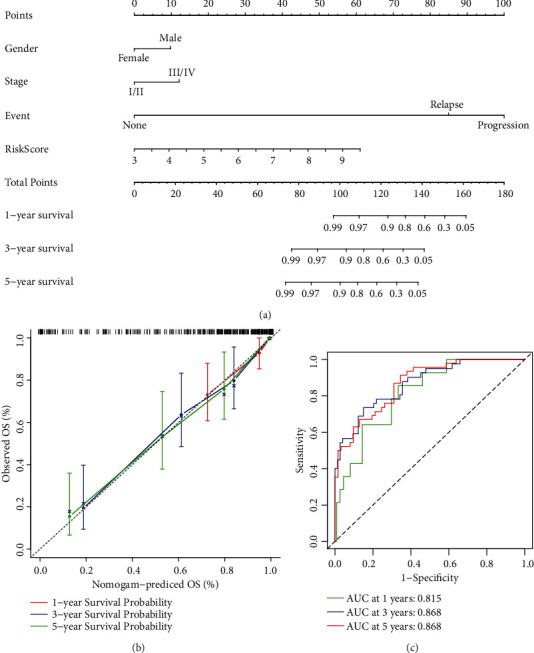
Construction and validation of the nomogram model. (a) Nomogram integrating the points of clinical characteristics and risk scores to predict the probability of 1-, 3-, and 5-year OS in pediatric patients with WT. (b) Calibration plots for assessing the discrimination ability of the nomogram model. (c) ROC curves for validating the predictive performance of the nomogram model.

According to the univariate Cox regression analysis in the independent prognostic analysis, clinical features including gender, stage, and event, and risk scores were involved to construct an effective nomogram model for predicting the survival rates of 1-, 3-, and 5-year in pediatric patients with WT (Figure 5(a)). Through obtaining the corresponding scores of each variable and summing up these scores, the predicted survival rates would be calculated in pediatric patients with WT. The calibration plots showed good consistency between the actual probabilities and the estimated probabilities at 1-, 3-, and 5-year, as shown in Figure 5(b). Moreover, to assess whether the nomogram model has a credible prognostic performance, the ROC curves were calculated and showed that AUCs were 0.815, 0.868, and 0.868 at 1-, 3-, and 5-year, respectively (Figure 5(c)), and *C*-index was 0.83 (95% CI: 0.78-0.88). The above assessment showed our nomogram model had a good robustness.

### 3.5. Functional Enrichment Analysis and Tumor Microenvironment Analysis

**Figure 6:**
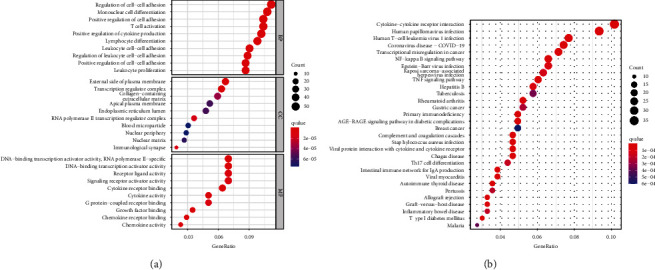
Function and pathway analysis based on DE-IRGs. (a) GO analysis is based on biological process (BP), cellular component (CC), and molecular function (MF). (b) KEGG analysis for the pathway.

**Figure 7:**
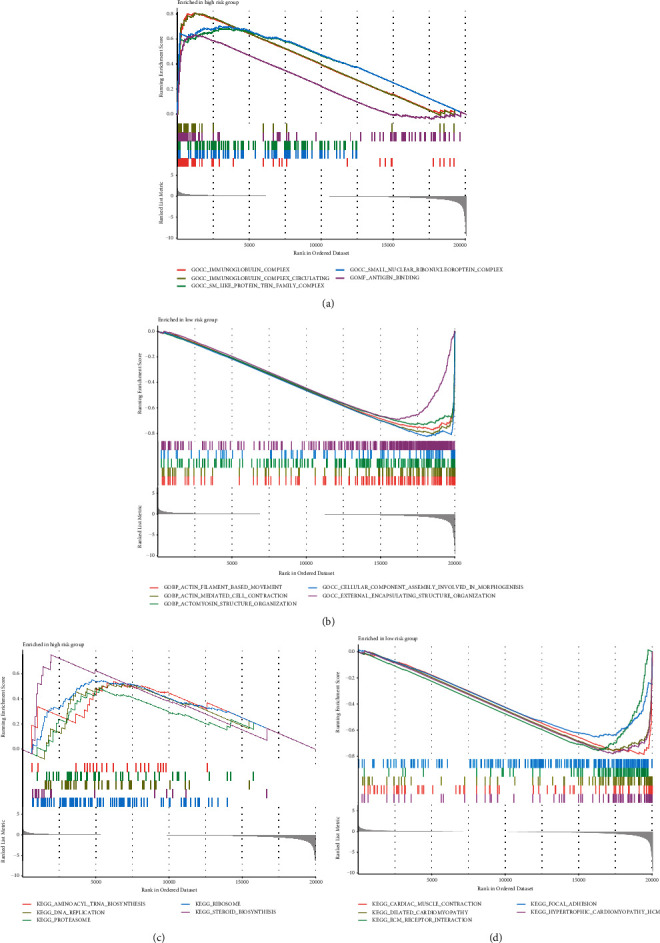
Function and pathway analysis for WT patients between high- and low-risk groups by GSEA analysis. (a, b) The GSEA analysis is based on GO for high-risk (a) and low-risk (b) groups. (c, d) The GSEA analysis is based on KEGG for high-risk (c) and low-risk (d) groups.

GO and KEGG were applied to further elucidate the biological functions and pathways in DE-IRGs between 125 tumor samples and 6 control samples. Besides, GSEA analysis was applied between low- and high-risk groups, as shown in Figure 6. For the GO analysis, the DE-IRGs among pediatric patients with WT mainly focused on the regulation of immune-related cells in biological process (BP), plasma membrane and transcription regulator complex in cellular component (CC), transcription activator activity, and receptor-ligand activity in molecular function (MF), as shown in Figure 6(a). The KEGG analysis about DE-IRGs in all pediatric patients with WT revealed that these DE-IRGs were enriched in immune-related pathways, cytokine−cytokine receptor interaction, and processing of a lot of diseases about infection, immunity, and tumor, such as human papillomavirus infection, COVID-19, primary immunodeficiency, and gastric cancer (Figure 6(b)). Above all, these DE-IRGs were also found the enriched behavior in the information-related biological processes based on GO and KEGG, such as leukocyte proliferation, regulation of leukocyte cell-cell adhesion, NF-kappa B signaling pathway, TNF signaling pathway, and regulation of inflammatory response (Table S2 and S3). This feature suggested the feasibility of establishing an inflammation-related prognosis model in WT patients. Furthermore, GSEA analysis was applied to describe these biological functions and pathways in all genes of WT patients between high- and low-risk groups. Based on GO gene sets in GSEA analysis, the high-risk group focused on immune-related processes, while the low-risk group focused on actin-related processes (Figures 7(a) and 7(b)). Besides, our study also found that the regulation of inflammatory response is more active in the low-risk group (Table S4). The high-risk group mainly participated in DNA replication, aminoacyl, and steroid biosynthesis, while the low-risk group was associated with the processing of cardiomyopathy, cardiac muscle contraction, and adhesion pathway, as shown in the KEGG gene set analysis from GSEA analysis (Figures 7(c) and 7(d)).

**Figure 8:**
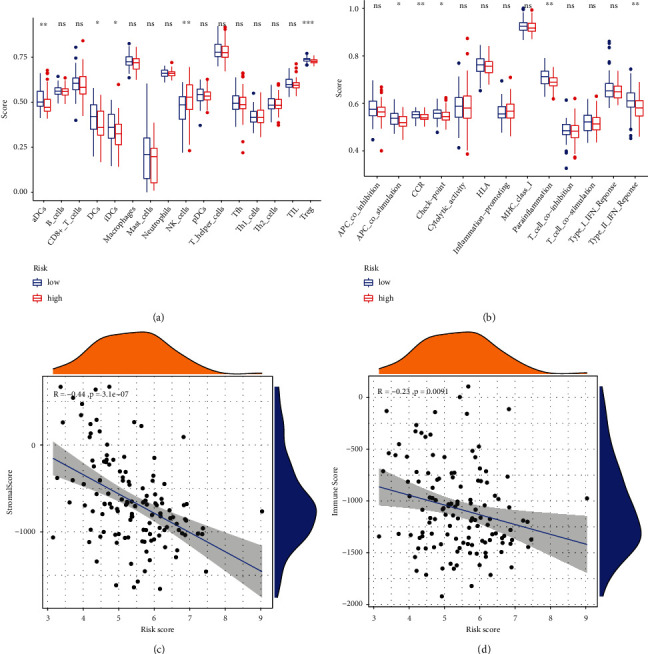
The differences between immune infiltration and tumor microenvironment analysis. (a, b) The results of ssGSEA to compare the differences of immune infiltration between high- and low-risk groups in pediatric patients with WT. ^∗^*p* < 0.05, ^∗∗^*p* < 0.01, ^∗∗∗^*p* < 0.001; ns: no significance. (c) Stromal score of TME plotted for showing the correlation between the content of stromal cells and risk scores. (d) The immune score of TME was plotted for showing the correlation between the content of immune cells and risk scores.

Involving the differences between 16 immune-related cells and 13 immune-related pathways, the ssGSEA analysis was performed to assess the activity of immune infiltration in the low- and high-risk groups (Figures 8(a) and 8(b)). In pediatric patients with WT, the low-risk group generally had higher levels of infiltration of immune cells than the high-risk group, especially of activated dendritic cells (aDCs), dendritic cells (DCs), regulatory T (Treg) cells, and induced dendritic cells (iDCs), while only natural killer (NK) cells were enriched highly in the high-risk group. All significant immune-related pathways were more enriched in the low-risk group, including parainflammation, APC_co_stimulation, CCR, check-point, and Type_II_IFN_Reponse. The stromal and immune scores were applied to understand the status of stromal cells and immune environment in pediatric patients with WT among different risk scores. The stromal and immune scores decreased significantly with the increase in risk score, which demonstrated that pediatric patients with WT with high risk have lower levels of stromal and immune cells than those with low risk (all *p* < 0.05) (Figures 8(c) and 8(d)).

### 3.6. Drug Sensitivity Analysis

**Figure 9:**
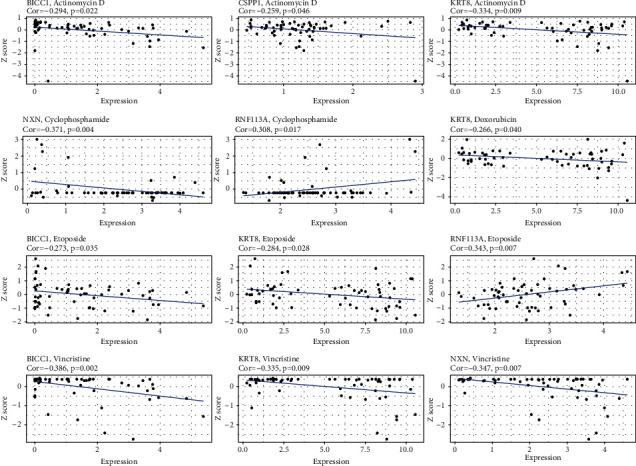
Drug sensitivity analysis founded on the IRGs signature in pediatric patients with WT.

To explore the underlying relationship between inflammatory signatures and the treatment of WT patients, the relevance between 7-IRGs signatures and 119 antitumor drugs approved by FDA were screened in Table S5, of which 5 drugs applied in chemotherapy for pediatric patients with WT in the National Comprehensive Cancer Network (NCCN) were shown in Figure 9. The drug-resistant of actinomycin D, etoposide, and vincristine increased with the upregulation of BICC1 and KRT8, while the drug sensitivity of cyclophosphamide and etoposide increased with the upregulation of RNF113A (Figure 9). In addition, NXN is insensitive to cyclophosphamide and vincristine, CSPP1 is insensitive to actinomycin D, and KRT8 is insensitive to doxorubicin (all *p* < 0.05). More importantly, the more relevant and potential correlations were found (|cor| >0.5, *p* < 0.05), of which KRT8 is insensitive to pipamperone and carmustine, RNF113A is sensitive to carmustine, and BICC1 has a sensitivity with erlotinib (Table S5).

## 4. Discussion

WT is the most common type of pediatric kidney cancer. At present, the chief treatment directions of pediatric patients with WT are to reduce drug toxicity in low-risk patients and improve the outcome of high-risk patients based on risk classification management, and the use of biomarkers to improve risk stratification and new targeted therapies has become an important research direction [24]. Exploring the close relationship between inflammation and immunity may promote the treatment direction in WT patients [10, 25]. One study supports the above view that the progression of the inflammatory marker COX-2 can activate the inflammatory microenvironment and inhibit the immune response to escape immunosurveillance in the inflammatory environment of WT, which makes COX-2 become a probable treatment target in WT patients [13]. For further exploration, the application of inflammatory biomarkers needs further evaluation in the prognosis of WT patients.

In this study, we first studied the mRNA profiles based on 1137 IRGs from GSEA and TARGET databases in pediatric patients with WT. Identifying these IRPGs between DE-IRGs and OS-related genes was further applied in the LASSO regression and multivariate Cox regression to explore more appropriate inflammation-related signatures and generate an optimal prognostic IRGs model. In addition, based on the independent prognostic analysis including clinical characteristics and risk scores, a novel prognostic nomogram model was constructed for further comprehensive analysis assessing prognosis in pediatric patients with WT, of which the endpoint event, stage, and risk scores were identified as the significant factors predicting the prognosis. The function enrichment analysis revealed the differences of potential pathways and immune infiltration between high- and low-risk groups. In addition, stromal scores and immune scores of TME relevance analysis were beneficial to show the correlation of risk scores and WT development. Drug sensitivity analysis has a potential role in studying regulatory targets to reduce the resistance of chemotherapeutic drugs in pediatric patients with WT.

The prognostic model developed on seven IRGs (BICC1, CSPP1, KRT8, MYCN, NELFA, NXN, and RNF113A). The above seven prognostic IRGs have not been reported in 32 confirmative biomarkers of WT, thereby IRGs as prognostic signature may be novel and potential biomarkers in diagnostic tests, potential therapies, and prognostic assessment [5]. KRT8 (keratin 8) has been identified as having its mutations associated with the occurrence of inflammatory diseases such as chronic liver disease, pancreatitis, and inflammatory bowel disease [26–29]. MYCN was found that its amplification was associated with poor prognosis and relapse in WT patients [30]. MYCN-amplification can cause several negative events in the tumor microenvironment and inflammatory regulation of neuroblastoma, such as damage to the infiltration and activation of T cells, more vascularized tumor, and downregulation of MHC I [31, 32]. NELFA is one of the components of the four-subunit NELF complex [33]. A study by Yu et al. found that NELF positively regulated the genes' transcription and processes of macrophage-mediated inflammation by inhibiting AP-1-dependent expression of IL-10 and facilitating IL-6 production [34]. Furthermore, macrophages are a common infiltration cell in chronic inflammation promoting tumorigenesis [8, 35]. In addition, BICC1 expression was positively and strongly correlated with immune cells and macrophages in gastric cancer [36]. Upregulation of IRGs and macrophage markers were found in transgenic mice with high nucleoredoxin (NXN) expression that can promote adipogenic differentiation by restraining the Wnt/*β*-catenin signaling pathway [37]. Furthermore, inhibiting the Wnt/*β*-catenin signaling pathway by dietary polyphenols can hinder the occurrence and development of chronic inflammation [38]. CXCR4 recruits inflammatory cells and is degraded by the overexpression of RNF113A and may be helpful to resist immunosuppression [8, 39, 40]. CSPP1 has been identified to inhibit tumor cell migration, proliferation, formation, and invasion when it was decreased and related to PI3K/Akt signaling pathway that can reduce inflammation by downregulating the degranulation of mast cells [8, 41–44]. In our study, MYCN, NELFA, and RNF113A were identified as the risk genes (coef > 0 and HR > 1), while BICC1, CSPP1, KRT8, and NXN as the protecting genes (coef < 0 and HR < 1) in the prognosis risk model based on IRGs in pediatric patients with WT (Table S6). Of note was how these genes interact and influence needs further investigation and experiment in the inflammatory metabolism and development of WT.

Inflammation cannot leave the immune process in WT patients [25]. GO, KEGG, and GSEA were applied based on the DE-IRGs and two risk groups, which demonstrated that the function and pathway of IRGs were mainly enriched in inflammation pathways, immunity pathways, and the pathways of many other diseases. There is some definite evidence that the NF−kappa B signaling pathway, TNF signaling pathway, Th17 cell differentiation, and T cell activation in the outcomes of functional enrichment analysis participated in the occurrence and development of inflammation, especially immune function and pathway [45, 46]. Similarly, enrichment of immunoglobulin function and the antigen-binding process was observed in the high-risk group. The results of our TME analysis suggested that a higher risk for pediatric patients with WT leads to a lower immune score, which indicated that high-risk patients with WT exist in the immunosuppressive tumor microenvironment. This phenomenon was also observed in the ssGSEA analysis, where the high-risk groups had relatively low immune infiltration. We further postulate that appropriate reactivation and enhancement of immune function may be beneficial for pediatric WT therapy. Furthermore, no significant treatment efficacy was observed with the use of an immune checkpoint inhibitor targeting PD-L1 for pediatric tumors, including WT patients [47]. We found that NK cells were enriched higher in high-risk patients with WT. However, other evidence showed that the function of NK cells in WT can be undermined to favor tumor escape and generation of immunosuppressive tumor microenvironment by blastemal and epithelial tumor components of WT [48]. Activation of NK cells can kill WT primary cells, but M2 macrophages can damage NK cells, and the combination of checkpoint inhibitors to inhibit macrophage recruitment and activate NK cells is expected to become an effective strategy for the treatment of WT patients [48, 49]. Our study also found that the regulation of inflammatory response is more active in low-risk pediatric patients with WT. Furthermore, inhibiting macrophage recruitment is also an effective strategy to treat chronic inflammation to antagonize tumorgenesis [8]. Through secreting inflammatory factors, pDCs and Tregs can favor immunosuppression and tumor processing in WT [13]. Therefore, regulating the inflammatory metabolic process is a potential pathway to improve the efficacy of immunotherapy in pediatric patients with WT.

Founded on the microenvironment of inflammatory response in WT patients, inflammatory markers could favor to the development of new treatments [25]. In our study, the relationship between 7-IRGs signature and drug activity in drug sensitivity analysis indicated that targeted regulation of these signatures can help to improve the objective drug response and the discovery of new drugs to treat WT, which further showed the value of 7-IRGs signature in the treatment of pediatric patients with WT. Actinomycin D, etoposide, vincristine, cyclophosphamide, and doxorubicin are regarded by NCCN as the first-line chemotherapy for pediatric patients with WT in 2021 [1]. Besides, there are some potential drugs in pediatric patients with WT. For instance, even though irinotecan was used to treat colorectal, pancreatic, and lung cancer at present, irinotecan as a potential drug was effective in treating high-risk metastatic DAWT with a high response rate (79%) through the combination with vincristine [2, 50]. In the drug sensitivity analysis, irinotecan has a significant positive correlation with RNF113A and NELFA upregulated in WT samples, as shown in Table S5. And as mentioned earlier, upregulation of RNF113A can resist immunosuppression. Even if carboplatin was not included in the treatment regimen of NCCN, its safety and effectiveness based on combination with ifosfamide and etoposide had been demonstrated in high-risk patients with WT, especially after nephrectomy [51]. When actinomycin D is not available or hard to tolerate for WT patients, carboplatin can become alternative drug [52]. In our study, carboplatin sensitivity increased with upregulation of RNF113A or MYCN and decreased with upregulation of KRT8, as shown in Table S5. We also found that upregulation of KRT8 is related to drug resistance in WT patients. In previous studies, upregulation of KRT8 can enhance the resistance of cisplatin by inhibiting the AKT pathway [53], and decrease the sensitivity of mitoxantrone [54]. Therefore, according to the degree of different risk scores and regulation of 7-IRGs signature in pediatric patients with WT, the selection of appropriate or potential drugs may help to improve the efficacy of treatment. Notably, although our study offers new insight into the therapeutic agents for WT patients based on the 7-IRGs signature, it still needs to be verified through clinical trials of drugs.

Some limitations cannot be avoided in this study. First, the prognostic values of the seven-IRGs signature were not be validated using another database owing to the lack of other relevant database including WT samples. Second, the sample size of pediatric patients with WT in the TARGET database was a comparatively small resulting in the bias of the prognostic model possibly. And then, the number of control samples (*n* = 6) may lead to bias in the stability of screening DE-IRGs. Ultimately, our study was not involved in experimental verification and other data types including lncRNA and DNA methylation. Our study only included the expression profile of the protein-encoded RNA.

## 5. Conclusions

In summary, our study demonstrated that IRGs were correlated with OS, immune biological processing, and chemotherapy drugs in pediatric patients with WT. Through identifying 7-IRGs signature, we developed a novel effective risk model and a prognostic nomogram model with good robustness. Besides, regulating the immune process of WT based on the inflammatory response can contribute to improving the efficacy of WT patients. The chemotherapy drug sensitivity of WT patients may be controlled, and the potential drugs of treatment can be excavated by regulating the 7-IRGs signature. Our study provides a new insight to the development and treatment in pediatric patients with WT based on the inflammatory response. However, the idiographic mechanisms among IRGs, drug sensitivity, and the prognosis of pediatric patients with WT still need further study by experiment.

## Figures and Tables

**Table 1:** The clinical characteristics of high- and low-risk groups in pediatric patients with WT.

Covariates	Group	Total (*n* = 125)	High-risk (*n* = 62)	Low-risk (*n* = 63)	*p* value
Gender	Female	71 (56.8%)	34 (54.84%)	37 (58.73%)	0.661
	Male	54 (43.2%)	28 (45.16%)	26 (41.27%)	

Age	<6	93 (74.4%)	49 (79.03%)	44 (69.84%)	0.316
	≥12	5 (4%)	3 (4.84%)	2 (3.17%)	
	6~12	27 (21.6%)	10 (16.13%)	17 (26.98%)	

Event	None	27 (21.6%)	10 (16.13%)	17 (26.98%)	0.007
	Progression	7 (5.6%)	7 (11.29%)	0 (0%)	
	Relapse	91 (72.8%)	45 (72.58%)	46 (73.02%)	

Stage	I/II	65 (52%)	27 (43.55%)	38 (60.32%)	0.061
	III/IV	60 (48%)	35 (56.45%)	25 (39.68%)	

Histologic	DAWT	42 (33.6%)	24 (38.71%)	18 (28.57%)	0.230
	FHWT	83 (66.4%)	38 (61.29%)	45 (71.43%)	

## Data Availability

The RNA-sequencing expression profile and relevant clinical information of pediatric patients with Wilms tumor were downloaded from the TARGET database (https://ocg.cancer.gov/programs/target). The inflammation-related genes can be obtained from GSEA molecular signatures database (http://www.gsea-msigdb.org/gsea/msigdb). The *Z* scores and expression profile of drug sensitivity analysis were downloaded from the CellMiner database (https://discover.nci.nih.gov/cellminer/home.do). These databases used in this study were publicly accessed and obtained.

## Supplementary Materials

Supplementary Materials Supplementary Table 1 (Table S1) is additional information about inflammation-related genes. Supplementary Table 2 (Table S2) is the complete result of GO analysis based on DE-IRGs. Supplementary Table 3 (Table S3) is the complete result of KEGG analysis based on DE-IRGs. Supplementary Table 4 (Table S4) is the result of GSEA based on GO. Supplementary Table 5 (Table S5) is the result of drug sensitivity founded on the correlation of 7-IRGs signature. Supplementary Table 6 (Table S6) is the coefficient and hazard ratio of prognostic model based on 7-IRGs.
